# A Self-Regulation eHealth Intervention to Increase Healthy Behavior Through General Practice: Protocol and Systematic Development

**DOI:** 10.2196/resprot.4835

**Published:** 2015-12-22

**Authors:** Jolien Plaete, Ilse De Bourdeaudhuij, Maite Verloigne, Anke Oenema, Geert Crombez

**Affiliations:** ^1^ Department of Movement and Sport Sciences Ghent University Ghent Belgium; ^2^ School for Public Health and Primary Care (CAPHRI) Department of Health Promotion Maastricht University Maastricht Netherlands; ^3^ Department of Experimental-Clinical and Health Psychology Ghent University Ghent Belgium

**Keywords:** intervention mapping protocol, physical activity, fruit intake, vegetable intake, eHealth, self-regulation, general practice

## Abstract

**Background:**

Chronic diseases are the principal cause of morbidity and mortality worldwide. An increased consumption of vegetables and fruit reduces the risk of hypertension, coronary heart disease, stroke, and cancer. An increased fruit and vegetable (FV) intake may also prevent body weight gain, and therefore indirectly affect type 2 diabetes mellitus. Insufficient physical activity (PA) has been identified as the fourth leading risk factor for global mortality. Consequently, effective interventions that promote PA and FV intake in a large number of people are required.

**Objective:**

To describe the systematic development of an eHealth intervention, MyPlan 1.0, for increasing FV intake and PA.

**Methods:**

The intervention was developed following the six steps of the intervention mapping (IM) protocol. Decisions during steps were based upon available literature, focus group interviews, and pilot studies.

**Results:**

Based on needs assessment (Step 1), it was decided to focus on fruit and vegetable intake and physical activity levels of adults. Based on self-regulation and the health action process approach model, motivational (eg, risk awareness) and volitional (eg, action planning) determinants were selected and crossed with performance objectives into a matrix with change objectives (Step 2). Behavioral change strategies (eg, goal setting, problem solving, and implementation intentions) were selected (Step 3). Tablet computers were chosen for delivery of the eHealth program in general practice (Step 4). To facilitate implementation of the intervention in general practice, GPs were involved in focus group interviews (Step 5). Finally, the planning of the evaluation of the intervention (Step 6) is briefly described.

**Conclusions:**

Using the IM protocol ensures that a theory- and evidence-based intervention protocol is developed. If the intervention is found to be effective, a dynamic eHealth program for the promotion of healthy lifestyles could be available for use in general practice.

## Introduction

Chronic diseases, such as cardiovascular disease, type 2 diabetes, and cancer, are the principal cause of morbidity and mortality worldwide, representing 68% of all deaths [1]. An increased consumption of vegetables and fruit reduces the risk of hypertension, coronary heart disease, stroke, and cancer. Furthermore, an increased fruit and vegetable (FV) intake may also prevent body weight gain, and therefore indirectly affect type 2 diabetes mellitus [2]. Insufficient physical activity (PA) has been identified as the fourth leading risk factor for global mortality [3]; it accounts for 6% of all deaths per year, and accounts for 21-25% of breast and colon cancers, 27% of diabetes, and 30% of ischemic heart disease worldwide [4,5]. Consequently, effective interventions that promote PA and FV intake in a large number of people are required. Reviews have shown that eHealth interventions are effective in changing PA and FV intake [6,7]. In eHealth interventions, information and communication technologies are used to improve or enable health and health care [6]. eHealth interventions allow a personalized approach at a relatively low cost by making use of interactive, computerized technologies [6,8], and have several advantages: reduced personal demands, consistency over time, increased interactivity and flexibility, automated data collection, and more honest self-report. Most of these interventions are delivered through the Internet only [6,9,10]. Although a large number of people can be reached through the Internet, the percentage of individuals who start with an Internet-delivered intervention is low, and sustained use is even lower [11]. Reach and use of eHealth interventions can be enhanced by the provision of additional support [11]. General practitioners (GPs) may be influential in supporting patients by providing extra information when implementing the intervention [12-14]. The reach and sustained use of eHealth interventions may also be increased by using computer-tailored feedback and facilitating goal setting and self-monitoring, as well as by incorporating email and short message service (SMS) text message reminders as behavior change methods into eHealth interventions [15-17]. Although eHealth interventions have been shown to be effective, effect sizes of eHealth interventions that target PA and dietary behavior are small [16,18]. Computer-tailored feedback merely targets variables that primarily address the adoption of an intention to change (eg, attitude or social norm), hence leaving many individuals in an intention-behavior gap. It is also important to address this gap by addressing postintentional factors (eg, action planning and problem solving). A self-regulation perspective may be well suited to integrate both pre- and postintentional processes, and to develop interventions that guide individuals during all phases of behavior change [19,20]. Self-regulation techniques can empower adults and allow them to make more autonomous decisions about their own health behavior [19,21,22]. Self-regulation is a goal guidance process which occurs in a motivational and volitional phase [19]. During the motivational phase (ie, goal selection, goal setting, and representation), participants become aware of risks, form intention-to-change behavior, and set goals to change their behavior. In the volitional phase (ie, active goal pursuit, goal attainment, and maintenance or goal disengagement), participants make action plans, engage in goal pursuit, and maintain or adapt their goals [19].

In this study, a dynamic eHealth intervention, MyPlan 1.0, was developed that targets self-regulation processes to increase PA and FV intake. To enhance reach and use, the intervention will be implemented in general practice. To ensure that MyPlan 1.0 is theory and evidence based, as well as feasible for implementation in general practice, the intervention mapping (IM) protocol was used as the planning model for the intervention [23]. IM facilitates effective decision making by formalizing the development process of the intervention in six steps [23]. Via the IM protocol, researchers are guided in selecting target behaviors (Step 1), specifying intervention goals (Step 2), choosing intervention strategies (Step 3), and developing intervention tools and programs (Step 4). IM also involves the planning of the implementation (Step 5) and the evaluation (Step 6). This paper describes the theoretical considerations and decisions made during each step of the IM protocol. This resulted in a detailed intervention description, which provides insight into the design and different components of the intervention, and will help planners to identify techniques and replicate the different intervention components [24].

## Methods

The IM protocol consists of the following six steps: (1) needs assessment, (2) development of matrices of change, (3) selection of theory-based methods and practical applications, (4) description of the program production, (5) development of a program adoption and implementation plan, and (6) completion of an evaluation plan [23].

In Step 1, a planning group was established to ensure that the intervention targets important factors to increase effectiveness and sustained used of the eHealth intervention. Based on the needs assessment, we also selected the target behaviors in Step 1.

In Step 2, we adopted self-regulation theories to determine the intervention content and to formulate performance objectives. Different statements were formulated about how participants may achieve the intervention goals. These statements are specific actions that have to be taken by participants and are called performance objectives [23]. Next, relevant and changeable determinants of the target behavior were selected in the second step. Finally, change objectives were formulated by stating what needs to be changed regarding a determinant in order to accomplish a performance objective.

In Step 3, theory-based methods that can modify the selected determinants to achieve the performance objectives were determined. Matching methods were selected based upon the results of systematic reviews that summarized the effectiveness of behavior change methods for healthy eating and physical activity interventions. We also took into account the summary list published by Bartholomew et al [23] and the taxonomy of behavior change techniques published by Abraham and Michie [24].

In Step 4, an intervention plan was developed, based on the selected methods and practical applications. Previous programs that were effective were used as examples [25-28]. Furthermore, a pretest of the intervention was conducted in Step 4 to identify possible elements of improvement and to evaluate the feasibility and user-friendliness of the intervention program.

In Step 5, the implementation of the intervention was planned. Support by general practitioners has been shown to improve the use and the effect of computer-tailored programs [11]. Therefore, GPs were involved in the implementation of MyPlan 1.0 in general practice. To this end, during Step 5, we conducted focus group interviews with GPs regarding the implementation.

The aim of this paper was to describe the intervention development. It is therefore not a research protocol of the trial, which is reported at ClinicalTrials.gov (trial registration number: NCT02211040). Therefore, in Step 6, we only specify the evaluation design and briefly describe the evaluation plan. We briefly describe the decisions made during each step of the IM protocol in the results section.

## Results

### Step 1: Needs Assessment

The planning group consisted of six researchers from different health disciplines—physical activity, nutrition, psychology, and primary health care—and leading GPs from the Belgian association of GPs, who are potential end users of the program. The core theories, methods, practical applications, implementation options, and evaluation strategies were discussed among this planning group.

Based on needs assessment, physical activity and fruit and vegetable intake were selected as target behaviors. Insufficient physical activity and unhealthy diet are two important risk factors of chronic diseases (eg, diabetes and ischemic heart diseases) and cancers (eg, breast and colon cancer) [1,29]. Adults are recommended to have 30 minutes of moderate-intensity aerobic PA 5 days per week, or to have 20 minutes of vigorous-intensity aerobic PA 3 days per week [30]. However, these recommendations are not reached by a large part of the population [5], nor by a majority of Belgian adults (62%) [31]. Therefore, it was decided to target physical activity levels in various subdomains (eg, activities at work, activities during leisure time, and active transports and sports).

Adults are recommended to consume at least five portions or 400 grams of fruit and vegetables a day, and data from the World Health Survey showed that 78% of the adult population consumed less than five portions of fruit and vegetables daily [32,33]. Western adults (ie, in Belgium, Luxembourg, France, Ireland, the Netherlands, and Great Britain) consume on average 129 grams of fruit and vegetable per day [2]. Furthermore, an evaluation of the gap between food-based dietary guidelines and the usual food consumption in Belgium indicated that fruit and vegetable consumption was significantly lower than recommended in a large part of the Belgian population. Of Belgian adults, 53% and 62% do not eat fruits and vegetables on a daily basis, respectively [34]. Therefore, we also decided to focus on fruit and vegetable intake as a dietary component of the intervention. After the needs assessment, the intervention goals were as follows: (1) to increase fruit and vegetable intake, and (2) to increase physical activity levels in Belgian adults (older than 18 years).

### Step 2: Performance Objectives, Determinants, and Change Objectives

The performance objectives for the target PA are shown in Table 1, and those for fruit and vegetable intake are shown in Multimedia Appendix 1. For example, the first performance objective for PA is “Adults recognize the importance of increasing PA levels.”

**Table 1:** Performance objectives for physical activity.

Phases		Performance objectives
**Motivational phase**		Goal selection, setting, and representation
	Performance objective 1	Adults recognize the importance of increasing physical activity levels
	Performance objective 2	Adults decide to change their physical activity levels and set physical activity goals
**Volitional phase**		Active goal pursuit
	Performance objective 3	Adults choose their own strategies to change their physical activity levels
	Performance objective 4	Adults start pursuing their physical activity goals
	Performance objective 5	Adults monitor and evaluate their physical activity levels
	Performance objective 6	Adults maintain or adapt their physical activity goals to a higher level
	Performance objective 7	Adults adapt their goals and strategies when they are unable to reach their initial goals

The health action process approach model of Schwarzer [20] was used to identify and categorize determinants within a self-regulation framework. This model has been successfully applied to predict fruit and vegetable intake [35,36] and physical activity [20,37-40]. The model categorizes determinants into two phases: a motivational phase and a volitional phase [20]. In the former phase, risk awareness, outcome expectancies, and preaction self-efficacy are determinants that influence intentions. After an intention is formed and goals are set, participants try to achieve their goals. In the volitional phase, action planning, coping planning, maintenance self-efficacy, and social support are determinants that influence actual changes in fruit and vegetable intake and physical activity levels. Maintenance and recovery self-efficacy are important determinants for participants to choose to maintain or adapt their goals, based on an evaluation of their behavior change [20].

All performance objectives, related change objectives, and determinants for the target behavior PA are shown in Multimedia Appendix 1. For example, to accomplish performance objective 2—“Adults decide to change their PA level and set PA goals in one or more subdomains”—change objective 2.5—“Adults identify for which PA goals they have the highest level of confidence”—describes what needs to be changed regarding the determinant *preaction self-efficacy*.

### Step 3: Selection of Theory-Based Methods and Practical Applications

In Tables 2 and 3, an overview of the methods and practical applications used in the intervention is given for the motivational and volitional phases, respectively. For example, the selected theoretical method *stating implementation intentions* corresponded with the determinants *action planning*, and *coping planning*.

To translate the methods into practical applications, we used study protocols of effective interventions [25-28,41-45]. We also used methods incorporated in an original program from our research group developed by Vandelanotte et al [27] and Spittaels et al [28]. This original program gave only feedback on motivational determinants (eg, intentions, attitudes, and knowledge). To effectively translate techniques that also target volitional determinants into practical applications, we used the programs of van Genugten et al [41], Walthouwer et al [25], and Springvloet et al [26]. For example, a practical application that was formulated by the method of *implementation intentions* to target *coping planning* was to let adults formulate a coping plan by formulating if/then plans (ie, implementation intentions). After the “if” is determined, selected difficult situations or barriers are stated. After the “then” is determined, selected solutions to overcome these difficult situations and barriers are stated (eg, *If* it is Monday evening and I am not in the mood for sports, *then* I call my friend to go to the aerobic lessons together).

**Table 2:** Methods and practical applications used in the intervention for the motivational phase.

Methods	Determinants	Practical applications
General information	Risk awareness	General information is provided in the form of short texts and slogans. In these texts and slogans, physical activity guidelines and health benefits of sufficient physical activity levels are highlighted.
	Outcome expectancies	Adults can read information about physical activity and select the information that they are interested in on a website. They can, for example, select to read information about positive outcomes due to sufficient physical activity levels or information about the benefits of increasing physical activity levels.
Monitoring, tailored feedback, and personal risk information	Risk awareness	After filling in a questionnaire about physical activity level, personal feedback is provided in which adults’ levels of physical activity are provided, as well as how these compare to the recommended level.
Tailored feedback and modelling	Preaction self-efficacy	The tailored feedback includes stories about peers who succeeded in increasing physical activity levels, also in difficult situations. For example, “Eric (40 years old) decided to be more physically active in his free time, by walking in the local park for 30 minutes, three times per week. When it was raining, Eric decided to go swimming instead of walking.”
Prompting identification of barriers and problem solving, and tailored feedback	Preaction self-efficacy	A predefined list of possible difficulties (barriers and risk situations) to increase physical activity level is provided and adults can select these difficulties that are applicable to them. Based on their answers, tailored information and tips for solutions to overcome the indicated barriers and risk situations are provided; adults can select those solutions to apply which they are confident about.

**Table 3:** Methods and practical applications used in the intervention for the volitional phase.

Methods	Determinants	Practical applications
Selecting hindering factors/barriers and solutions Implementation intentions	Action planning Coping planning	Adults can first select hindering factors and barriers out of a predefined list. When applicable hindering factors and barriers are not available in the list, participants also have the possibility to write down another factor or barrier in an open-ended format. Next, participants can select solutions out of a predefined list or write down another solution. Afterward, participants are stimulated to make action plans and coping plans by formulating if-then plans (ie, “implementation intentions”). After the “if,” a situation or the previously selected difficult situations or barriers are stated and after the “then” the selected action or solutions to overcome the difficult situations and barriers are stated (eg, If it is Monday evening and I am not in the mood for sports, then I call my friend to go to the aerobic lessons together). Adults can formulate this implementation intention plan in an open-ended question format on the website.
Goal setting	Action planning	A list with personal and relevant goals is formed based on previous answers; adults can select the goals to change that they are confident about.
Stating SMART^a^goals	Action planning	Adults are guided by questions to make a *specific*, *measurable*, *attainable*, *relevant*, and *time-bound* (SMART) action plan. For example, adults can formulate answers to questions on what they want to do (eg, increase physical activity by biking 20 minutes to work), how often (eg, three times per week), when (eg, Monday, Wednesday, and Friday), and when they want to start (eg, starting on Monday, July 7). After answering all the questions, the personal action plan and the if/then plan are automatically generated and sent by email to the participant.
Public commitment	Social support	Adults can choose to send their action plan to others (eg, family and friends) to ask them to support them and invite them to also make an action plan.
Prompt self-monitoring of behavior and prompt review of behavioral goals	Action planning Maintenance self-efficacy	Adults are asked to keep a record of their physical activity levels or fruit and vegetable intakes by one of the given suggestions (ie, personal paper agenda, mobile phone, Excel sheet, or online agenda). After the active goal pursuit was started, adults are also invited by email to report their behavior on the website. Periodic email reminders are sent to invite adults to fill out a questionnaire about the target behavior and their goals on the website. The results are compared with their previous behavior and goals, and iterative feedback is provided on the progress of behavior change.
Set tasks on a gradient of difficulty	Maintenance self-efficacy	When adults have attained their goals, they are invited to change the goal by reformulating a more attainable or more difficult goal or by setting additional goals.
Planning coping responses	Coping planning Maintenance self-efficacy	Adults are asked whether they experienced barriers while pursuing their goals. If so, they are invited to identify solutions to cope with the identified situations or barriers. Adults can again select solutions from a list that is generated based on the selected difficulties.
Prompt review of behavioral goals and personal feedback	Recovery self-efficacy	When people do not achieve their goals, people get personal feedback that informs them that relapse is normal. They are also advised to try again, to choose other strategies, or to adapt their goals to more attainable goals.

^a^SMART: specific, measurable, attainable, relevant, and time-bound.

### Step 4: Producing the Program and Materials

**Figure 1:**
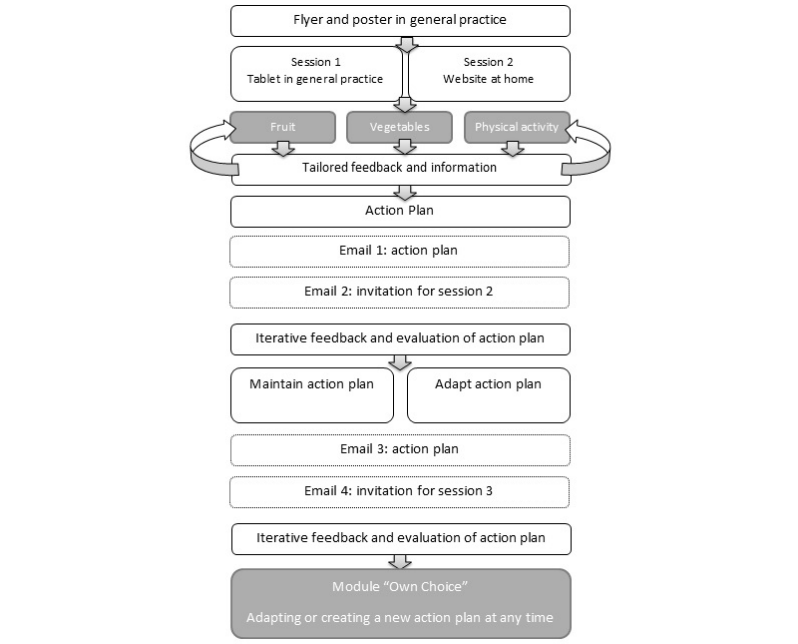
Overview of the intervention program.

MyPlan 1.0 was programmed in the freely available software LimeSurvey 2.0 [46]. In what follows, the intervention program is discussed; an overview of the intervention program is given in Figure 1. MyPlan 1.0 consisted of three modules—fruit, vegetables, and PA—that are available on a website and on a tablet computer. Participants can log in, choose a behavior of interest, and run through the first session of the chosen health behaviors.

**Figure 2:**
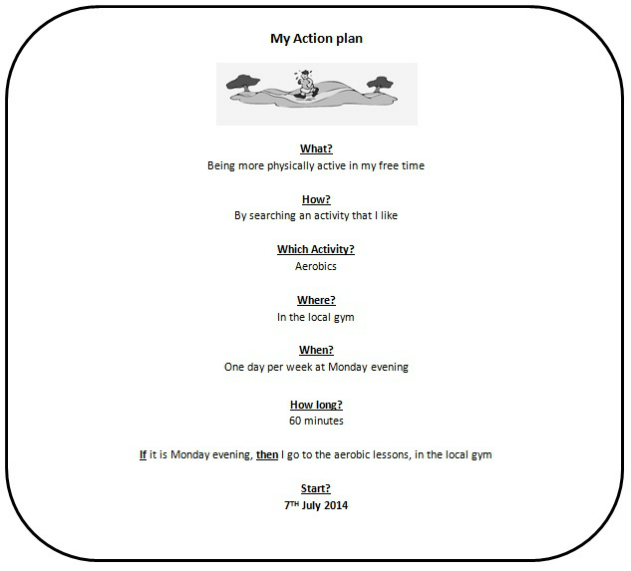
My Action plan: Example of an action plan for physical activity.

In the first session, people fill out a questionnaire and receive tailored feedback about how their behavior compares to the health norms. Next, adults can select and read more information about the behavior (eg, in relation to diseases and health) and can make an action plan. To make an action plan, adults first have to indicate whether they expect difficulties in changing their health behaviors. If so, adults can select or formulate barriers and reflect upon possible solutions to overcome the barriers. Afterwards, adults can make an if/then plan and an action plan by reading tips and filling in questions about how, when, and where they will act on their behavior. Based on the answers, an action plan is generated by the computer’s algorithm (see Figure 2).

It is proposed that participants monitor their behavior when they start pursuing their goals, and are invited to send their action plan to friends or family. When session 1 is completed, the action plan is emailed to the participant. A week after adults make their action plan, they receive an email with a link to the website where they can evaluate whether their formulated goal was accomplished. The current behavior is compared with the previous behavior and health goals, and iterative tailored feedback is provided. Based on this feedback, participants can decide to further pursue their goal, or to adapt their goal to a more difficult or more attainable goal. Participants also have the opportunity to reflect on encountered difficulties and to search again for solutions. The last session has a similar structure as session 2, and is available 1 month after completing session 1. At the end of session 3, patients are also referred to the module *Own Choice* on the website. This is an extra module, which participants can use at any time to adapt or to create an action plan for a behavior of their choice (eg, water intake). In this module, the same framework (ie, what, when, where, how many times, with who, and if/then) is used to enable adults to make a new action plan.

A further task in Step 4 is to test the feasibility, acceptability, and user-friendliness of the intervention [23,47]. Therefore, a specific study was conducted to address these issues, and its results are reported in a separate publication [48]. Briefly, 194 adults who used the MyPlan 1.0 intervention filled out an online questionnaire containing items about quality, user-friendliness, and applicability of the content and information architecture (ie, organization and delivery of the content) of the intervention. The results indicate that the program was generally well accepted, including for participants with a low educational level and for older participants. Nevertheless, to make the program more comprehensible for the different groups, the questions, answer options, and advice were made shorter and clearer [48]. To test the acceptability and user-friendliness of the tablet as a delivery mode, we conducted a thinking-aloud test with 40 adults. Most participants indicated that it was easy to use the intervention program on a tablet. Examples of comments that were reported were as follows: “text is too small to read on a tablet,” “moving from one page to another is too slow,” “and a pen to tick the answers would be useful.” Based on the comments, the intervention program was further adapted for appropriate use on the tablet.

### Step 5: Development of a Program Adoption and Implementation Plan

**Figure 3:**
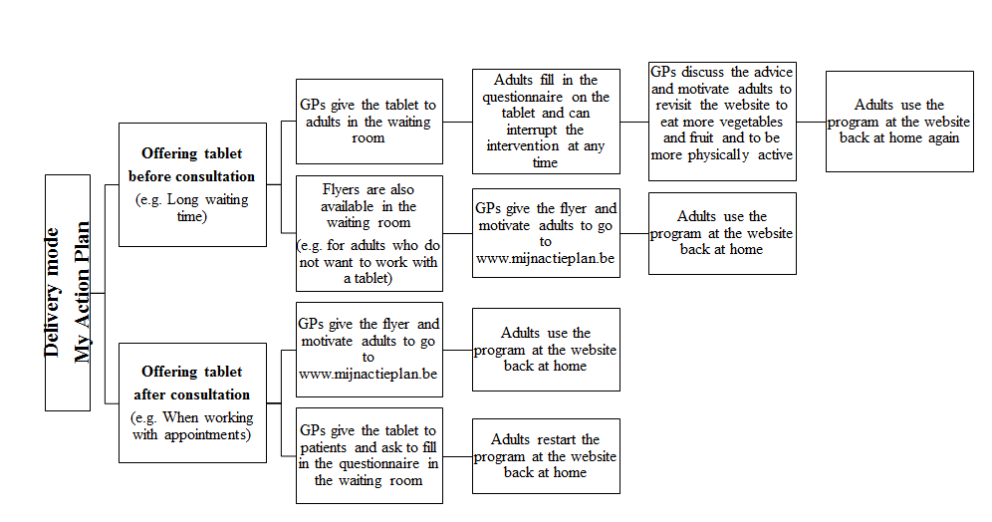
Decision tree for GPs: General practitioners can use the decision tree to decide on how to implement the intervention in general practice.

GPs who participated in the focus group interviews were positive about the use of a computer-tailored program that provides personal advice. GPs also appreciated that they did not need the expertise and time to compose personal advice for every patient, and may restrict their role to simply motivating and advising patients to use the intervention. However, doubts were raised on how to implement MyPlan 1.0 in general practice. By using tablets, MyPlan 1.0 patients can directly experience the use of the program and discuss their advice with their GP [49]. However, GPs indicated that in some situations it is not possible to use a tablet. For example, when there is not enough time, or when patients cannot work with a tablet. Therefore, it was decided to use a combination of flyers and tablets. Patients receive a flyer with a personal code in general practice, and can decide whether they start the program in general practice on a tablet or back at home at the website. On the flyer, it is also mentioned that participants can choose whether they want to discuss their personal advice and action plan with their GP in a following consultation. To briefly discuss the personal advice and action plans of patients, every participating GP received an information letter and attended a personal information session. In this session, GPs were instructed to emphasize the importance of personal and attainable health goals, rather than prescribing health recommendations and general information. GPs' opinions also differed about when to offer the intervention in general practice, indicating that different ways to use the program in general practice and solutions with different choices on how and when to use the program must be offered to GPs. Based on these results, it was decided to provide several modes of delivery that may be applicable in different workflow systems in general practice. Therefore, a decision tree (see Figure 3) with different choices on how to deliver the intervention in general practice was developed. In this way, GPs can autonomously decide which method is suitable for their own working system, for different patients, and for different circumstances. Other results of the focus group interviews are reported in more detail elsewhere [50].

### Step 6: Evaluation of the Program

**Figure 4:**
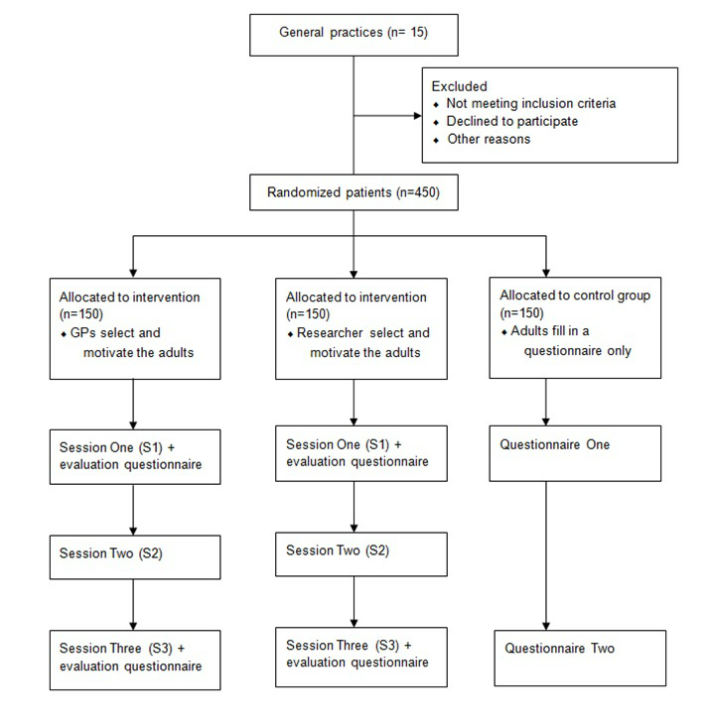
Design of the clustered quasi-experimental trial: A clustered quasi-experimental trial with three conditions will be used to evaluate the intervention. Group 1 is an intervention group recruited by a researcher, Group 2 is a control group recruited by a researcher, and Group 3 is an intervention group recruited by a GP.

A clustered quasi-experimental trial with three conditions will be used to evaluate the intervention (see Figure 4). We use a quasi-experimental design in which participants can be part of three different groups. First, a researcher recruits patients and randomly allocates them into an intervention (Group 1) or control group (Group 2). Next, a GP recruits patients into the other intervention group (Group 3). In both intervention groups, participants will not be randomly allocated to one of the behavior groups of the intervention (ie, PA, fruit, or vegetables), but participants can choose themselves for which behavior they want to complete the intervention. Group 1 is an intervention group in which researchers select and motivate adults to use the intervention by offering a flyer and/or tablet. In Group 3, the selection and motivation of adults will be randomly conducted by GPs in the waiting room by offering a flyer and/or tablet. Participants of both groups will receive a flyer with a personal code and can choose to start the intervention program in general practice on a tablet or at home on the website. Group 2 will be a waiting-list control group in which adults are randomly selected by a researcher. Participants in this control group only have to fill in a questionnaire and have no access to the computer-tailored feedback, action planning part, or to the evaluation in the follow-up modules. After completing the questionnaire at baseline and at the 1-month follow-up, the control group will also get access to the intervention modules.

In total, 30 adults will be selected in each of 15 general practices (n=450). First, a researcher will select 10 patients that will be allocated to the intervention group and 10 patients that will be allocated to the control group. Next, GPs will be asked to recruit another 10 patients to complete the intervention program. In this way, it can be evaluated whether GPs' involvement leads to more sustained use of the eHealth intervention, and higher levels of PA and FV intake. In both intervention groups, adults will be invited to complete session 1 either on a tablet in general practice or on their computer at home. Adults who do not use the tablet have to fill out a short questionnaire and leave their email address to be sent a reminder email to complete session 1 at home. After 1 week and 1 month of completing session 1, adults will receive an email to respectively start sessions 2 and 3. In the control group, adults will have to fill out a questionnaire at baseline in general practice or at home and at 1-month postintervention. To prompt adults to complete all questionnaires and sessions, reminder mails and SMS text messages will be sent. Inclusion criteria for participating in the study in both intervention and control groups are as follows: at least 18 years old, understand Dutch language, have an email address, and have access to the Internet. The outcome measures—increase in PA level, increase in FV intake, and self-regulation skills from baseline to postintervention—will be compared for the control and intervention conditions by conducting repeated measures multivariate analyses of variance (MANOVAs). Participant characteristics (ie, socioeconomic status [SES], age, sex, health status, and reaching health norms) will be compared at baseline. Characteristics that differ for the intervention and control groups will be added as covariates in further analyses. Furthermore, multilevel analyses will be conducted to take into account the clustering of participants into general practices.

## Discussion

Using IM increases the likelihood of developing an effective eHealth intervention and the transparency of intervention components, which makes replication possible for future researchers [24]. In the first step of the IM protocol we identified that PA levels and fruit and vegetable intake of adults were lower than recommended. In Step 2, the most important individual determinants for these low levels of PA and FV intake were determined and the objectives of the program were formulated. In Step 3, behavior change techniques were selected that are thought to affect these determinants and, hence, to achieve the stated objectives. In Step 4, the eHealth intervention MyPlan 1.0 was developed. Implementation strategies were selected in Step 5 and an implementation plan was made in Step 6.

Various eHealth interventions are based on motivational theories like the theory of planned behavior [51-53]. They most often target motivational determinants that are important during the early stages of behavior change, such as attitude and knowledge. However, interventions based upon theories of intentions are often more effective in changing intentions than in changing behavior [9,16], hence revealing the so-called intention-behavior gap. Our new eHealth program was partly based on a previous eHealth program developed by Vandelanotte et al [27] and Spittaels et al [28]. This original program was also based on the theory of planned behavior [54] and the transtheoretical model [55] and only gave feedback on motivational determinants (eg, intentions, attitudes, self-efficacy, and knowledge). The strategy of tailored feedback was further integrated to target the motivational determinants, but we also searched for new strategies such as goal setting, self-monitoring, and prompt review of goal progress to target volitional determinants.

Self-regulation has recently been considered as a preferred method to overcome the intention-behavior gap and thus to promote health behavior [19,40]. Therefore, the integration of the self-regulation skills in MyPlan 1.0 that target both motivational and volitional determinants was a particular strength of our study. Previous research showed more goal ownership in participants who set their own health goals. Participants who pursue their own health goals are also less likely to drop out of behavior change programs compared to participants who get prescribed health goals [56]. Another strength is that participants have the opportunity to choose between different target behaviors and can decide themselves what they would like to change. Choice is further incorporated into the program by letting participants select for themselves what information they want to read, which goals they want to set, and which strategies they want to use. It is expected that these features will increase goal ownership, which is known to lead to more internal motivation and empowerment, and more effective behavior change [19,21,22,25,57].

A further strength of our study is the comprehensive involvement of GPs in Step 5—implementation in general practice—of the IM protocol. To ensure the feasibility of the implementation in general practice, we involved GPs from the start of the development of the intervention. During focus group interviews, important barriers for the implementation of the intervention in general practice were reported. For example, the time burden for GPs when participating in preventive actions was of major importance. Therefore, an intervention in which the personal advice was provided by a computer program was well appreciated. MyPlan 1.0 can prompt GPs to motivate their patients to adopt a healthy lifestyle, but GPs are not expected to provide extensive preventive counseling. However, some GPs will make more of an effort than others to motivate patients. Therefore, in the evaluation study all participating GPs will be asked to motivate patients to use the intervention program to set personal and attainable health goals, rather than to prescribe health recommendations and general information. Also of importance is the creation of different choices about how and when GPs may implement the intervention. Therefore, a decision tree and a list of practical solutions to implement the intervention via tablets and flyers in general practice was generated.

In Step 6—evaluation—it will be investigated whether the direct involvement of GPs in the program matters. More specifically, we will evaluate whether GPs’ involvement leads to more sustained use of the intervention and higher levels of PA and FV intake. Also, multilevel analyses will be conducted to control for the clustering of participants into different general practices. Furthermore, it will also be important to evaluate the quality of participants’ action plans because previous research has shown that action plans of participants can be of poor quality [58].

Following the IM protocol is a complex and time-consuming enterprise [44,59]. Therefore, we suggest that future researchers search for existing study protocols that describe the development of interventions and integrate similar theories and methods that can be used as the basis for their intervention programs. In our study, existing protocols were used to translate self-regulation methods into practical applications in a computer-tailored program [25,26,41]. However, as target behaviors and contexts differ, it is important to further elaborate the different steps of the IM protocol for new interventions. Our program, for example, differs from the existing protocols in several ways. First, we used a program that made it possible to deliver tailored follow-up feedback in which changes in health behavior were mentioned and compared with health goals. This makes it possible to provide detailed tailored feedback on the behavior change process. Second, the delivery mode of our program is variable, as the program can be delivered via different channels, such as via the Internet and via tablets. The delivery via tablets made it possible to deliver MyPlan 1.0 in natural settings (ie, general practice). This leads us to the third important aspect on which our program deviates from other programs [25,26,41], namely, the possibility to integrate extra personal feedback by general practitioners. In conclusion, if MyPlan 1.0 is found to be effective, a new eHealth program for the promotion of PA and FV intake that can be applied by GPs will be available. Future research can focus on designing modules for other behaviors and on evaluating other methods and effective channels for implementation.

## Appendix

Multimedia Appendix 1 Performance objectives, their related change objectives, and their determinants for the target behaviors physical activity and fruit and vegetable intake.

## Abbreviations

CAPHRI: School for Public Health and Primary Care
FV: fruit and vegetable
FWO: Research Foundation-Flanders
GP: general practitioner
IM: intervention mapping
MANOVA: multivariate analysis of variance
PA: physical activity
SES: socioeconomic status
SMART: specific, measurable, attainable, relevant, and time-bound
SMS: short message service
