# Supplementary material for: A Self-Regulation eHealth Intervention to Increase Healthy Behavior Through General Practice: Protocol and Systematic Development
Source: JMIR Res Protoc. 2015 Dec 22;4(4):e141. doi: 10.2196/resprot.4835 (PMC4704890; doi:10.2196/resprot.4835)
Supplement: Multimedia Appendix 1 [file resprot_v4i4e141_app1.pdf]

Performance objectives and their related change objectives and determinants for the target behaviour PA in the motivational phase

	**Motivational determinants**			**Volitional determinants**			
**Performance objectives (POs)**	**Risk awareness**	**Outcome Expectancies**	**Pre-action self-efficacy**	**Action planning**	**Coping self-efficacy**	**Recovery self-efficacy**	**Social support**
**Goal selection, setting and representation**							
**PO1** **Adults recognize the importance of increasing PA levels**	CO 1.1 Adults are aware of their own PA level<br><br>CO 1.2 Adults explain the health norms of PA<br><br>CO 1.3 Adults compare their PA level with the health norm	CO 1.5 Adults describe that changing their PA level will help to be healthy, feel better and to prevent chronic diseases in the long term<br><br>CO 1.4 Adults list the possible consequences of their PA level when they do not reach health norms					
**PO2** **Adults decide to change their PA level and set PA goals in one or more subdomains**	CO 2.1 Adults are aware that improving PA level by pursuing attainable health goals can improve their health	CO 2.4 Adults summarize clear and reachable PA goals	CO 2.5 Adults identify for which PA goals they have the highest level of confidence<br><br>CO 2.6 Adults express				CO 2.7 Adults identify for which PA goals they will receive most social support

	status<br><br><u>CO 2.2</u><br>Adults explain that they can change their PA level in different subdomains (work, free time, household activities and active transport)<br><br><u>CO 2.3</u><br>Adults express which PA subdomain offers most possibilities for improvement		confidence that they are capable to select their own PA goals				

Performance objectives and their related change objectives and determinants for the target behaviour FV intake in the motivational phase

	Motivational determinants			Volitional determinants			
**Performance objectives (POs)**	**Risk awareness**	**Outcome Expectancies**	**Pre-action self-efficacy**	**Action planning**	**Coping self-efficacy**	**Recovery self-efficacy**	**Social support**
**Goal selection, setting and representation**							
**PO1** **Adults recognize the importance of increasing their FV intake**	CO 1.1 Adults are aware of their own FV intake<br><br>CO 1.2 Adults explain the health norms of FV intake<br><br>CO 1.3 Adults compare their FV intake with the health norm	CO 1.5 Adults describe that increasing their FV intake will help to prevent chronic diseases<br><br>CO 1.4 Adults list the possible consequences of insufficient FV intake					
**PO2** **Adults decide to increase their FV intake and set FV intake goals in one or more subdomains**	CO 2.1 Adults are aware that increasing their FV intake by pursuing attainable health goals can improve their health	CO 2.4 Adults summarize clear and reachable FV intake goals	CO 2.5 Adults identify for which FV intake goals they have the highest level of confidence<br><br>CO 2.6 Adults express				CO 2.7 Adults identify they will receive social support for their FV intake goals

	status		confidence that they are capable to select their own FV intake goals				

Performance objectives and their related change objectives and determinants for the target behaviour PA in the volitional phase

	Motivational determinants			Volitional determinants			
**Performance objectives (POs)**	**Risk awareness**	**Outcome Expectancies**	**Pre-action self-efficacy**	**Action planning**	**Coping self-efficacy**	**Recovery self-efficacy**	**Social support**
**Active goal pursuit**							
**PO3**<br>**Adults choose their own strategies to change their PA level**				CO 3.1<br>Adults indicate where they will reach their PA goal<br><br>CO 3.2<br>Adults describe how long they will change their PA level<br><br>CO 3.3<br>Adults describe how often they will change their PA level<br><br>CO 3.4<br>Adults describe when they will start to reach their PA goal and how they will achieve their PA goal at that moment	CO 3.6<br>Adults identify high risk situations to change their PA level<br><br>CO 3.7<br>Adults identify barriers to change their PA level<br><br>CO 3.7<br>Adults search for solutions to overcome identified barriers and high risk		CO 3.9<br>Adults inform other important persons about their PA goal and ask them for support

				CO 3.5<br>Adults identify needed resources to act on their PA goals	situations<br><br>CO 3.8<br>Adults express confidence that they can cope with difficult situations and barriers		
**PO4**<br>**Adults start pursuing their PA goals**				CO 4.1<br>Adults use their action plan and selected coping strategies to attain their PA goals	CO 4.2<br>Adults are confident to perform their PA goals (also in difficult situations)<br><br>CO 4.3<br>Adults identify new high risk situations, barriers and solutions while changing health behaviour		CO 4.4<br>Adults receive support when necessary
**PO5**<br>**Adults monitor and evaluate their PA level**	CO 5.1<br>Adults compare their PA level				CO 5.3<br>Adults express	CO 5.5<br>Adults have confidence to	

	with their PA goal<br><br><u>CO 5.2</u><br>Adults Recognize when behaviour change is (un)successful				confidence about how to evaluate changes in PA level<br><br><u>CO 5.4</u><br>Adults are able to make new coping plans where they are confident about	deal with deviations from the desired PA goal	
**Goal attainment and maintenance or goal disengagement**							
**<u>PO6</u>**<br>**Adults maintain or adapt their PA goals to a higher level**	<u>CO 6.1</u><br>Adults are aware of their PA change progress<br><br><u>CO 6.2</u><br>Adults recognize if they can adapt their goal to a higher goal		<u>CO 6.3</u><br>Adults express confidence to maintain their PA goal<br><br><u>CO 6.4</u><br>Adults express confidence that they can reach a higher goal	<u>CO 6.5</u><br>Adults adapt their action plan for a higher goal	<u>CO 6.6</u><br>Adults list solutions that are needed to overcome barriers to reach their higher PA goal	<u>CO 6.7</u><br>Adults feel able to pick up change after relapse	
**<u>PO7</u>**<br>**Adults adapt their goal and strategies when they are unable to reach their initial goal**	<u>CO 7.1</u><br>Adults are aware of their PA change progress<br><br><u>CO 7.2</u><br>Adults acknowledge		<u>CO 7.3</u><br>Adults express confidence that they can reach a more attainable goal	<u>CO 7.4</u><br>Adults adapt their action plan for a more attainable goal	<u>CO 7.5</u><br>Adults identify new high risk situations and barriers to reach their		

	that they should choose a more attainable goal to be capable to change their PA level				attainable health goal<br><br><u>CO 7.6</u><br>Adults select new Solutions and coping strategies to overcome identified barriers to reach their attainable health goal		

Performance objectives and their related change objectives and determinants for the target behaviour FV intake in the volitional phase

	Motivational determinants			Volitional determinants			
**Performance objectives (POs)**	**Risk awareness**	**Outcome Expectancies**	**Pre-action self-efficacy**	**Action planning**	**Coping self-efficacy**	**Recovery self-efficacy**	**Social support**
**Active goal pursuit**							
**PO3**<br>**Adults choose their own strategies to change their FV intake**				CO 3.1<br>Adults indicate where they will reach their FV intake goal<br><br>CO 3.2<br>Adults describe during which moments (breakfast, snack, dinner) they will change their FV intake<br><br>CO 3.3<br>Adults describe on how many days they will eat fruit and vegetables<br><br>CO 3.4<br>Adults describe how many portions of fruit and vegetables	CO 3.6<br>Adults identify hindering factors to increase their FV intake<br><br>CO 3.7<br>Adults search for solutions to overcome identified hindering factors to increase their FV intake<br><br>CO 3.8<br>Adults express confidenc e that they		CO 3.9<br>Adults inform other important persons about their FV intake goal and ask them for support

				they will eat<br><br><u>CO 3.5</u><br>Adults describe when they will start to reach their FV intake goal and how they will achieve their FV intake goal at that moment<br><br><u>CO 3.6</u><br>Adults identify needed resources to act on their FV intake goals	can cope with hindering factors to increase their FV intake		
**<u>PO4</u>**<br>**Adults start pursuing their FV intake goals**				<u>CO 4.1</u><br>Adults use their action plan and selected coping strategies to attain their FV intake goals	<u>CO 4.2</u><br>Adults are confident to perform their FV intake goals (also in difficult situations)<br><br><u>CO 4.3</u><br>Adults identify new high risk situations, barriers and solutions while		<u>CO 4.4</u><br>Adults receive support when necessary

					increasing FV intake		
<u>**PO5**</u><br>**Adults monitor and evaluate their FV intake**	<u>CO 5.1</u><br>Adults compare their new FV intake with their FV intake goal<br><br><u>CO 5.2</u><br>Adults Recognize when behaviour Change in FV intake is (un)successful				<u>CO 5.3</u><br>Adults express confidence about how to evaluate changes in their FV intake<br><br><u>CO 5.4</u><br>Adults are able to make new coping plans where they are confident about	<u>CO 5.5</u><br>Adults have confidence to deal with deviations from the desired FV intake goal	
**Goal attainment and maintenance or goal disengagement**							
<u>**PO6**</u><br>**Adults maintain or adapt their FV intake goals to a higher level**	<u>CO 6.1</u><br>Adults are aware of their FV intake change progress<br><br><u>CO 6.2</u><br>Adults recognize if they can adapt their FV intake goal to a higher goal		<u>CO 6.3</u><br>Adults express confidence to maintain their FV intake goal<br><br><u>CO 6.4</u><br>Adults express confidence that they can reach a higher FV intake goal	<u>CO 6.5</u><br>Adults adapt their action plan for a higher FV intake goal	<u>CO 6.6</u><br>Adults list solutions that are needed to overcome barriers to reach their higher FV intake goal	<u>CO 6.7</u><br>Adults feel able to pick up change after relapse of their FV intake goal	

**<u>PO7</u>**<br>**Adults adapt their goal and strategies when they are unable to reach their initial FV intake goals**	<u>CO 7.1</u><br>Adults are aware of their FV intake change progress<br><br><u>CO 7.2</u><br>Adults acknowledge that they should choose a more attainable FV intake goal		<u>CO 7.3</u><br>Adults express confidence that they can reach a more attainable FV intake goal	<u>CO 7.4</u><br>Adults adapt their action plan for a more attainable FV intake goal	<u>CO 7.5</u><br>Adults identify new high risk situations and barriers to reach their attainable FV intake goal<br><br><u>CO 7.6</u><br>Adults select new Solutions and coping strategies to overcome identified barriers to reach their attainable FV intake goal		
